# Removal of a congenital corneal dermoid through tumor excision and lamellar keratoplasty in a young child: A case report

**DOI:** 10.1097/MD.0000000000027981

**Published:** 2021-12-10

**Authors:** Yanyan Cui, Shan Yin, Xuewei Yin, Yonghui Liu, Bojun Zhao

**Affiliations:** a Shandong University of Traditional Chinese Medicine, Jinan, China; b Liaocheng People's Hospital, Liaocheng, China; c Department of Ophthalmology, Shandong Provincial Hospital Affiliated to Shandong First Medical University, Jinan, China.

**Keywords:** corneal dermoid, graft implantation, keratoconjunctivital tumor, keratoplasty, tumor resection

## Abstract

**Rationale:**

Corneal dermoids are a rare cause of corneal opacification, consisting of abnormal mesoblastic tissue surrounded by epithelium. Here, we describe the case of a 1-year-old child who had a congenital corneal dermoid in the left eye since birth; thus, the patient underwent tumor excision followed by keratoplasty.

**Patient concern:**

A 1-year-old girl was brought to the hospital by her parents, who had been noticing a mass on the surface of her left eyeball since birth. The patient had no other previous or concurrent disease nor family history for dermoids.

**Clinical findings:**

No abnormalities were present in the cornea and the anterior and posterior segments of the right eye. Eye movement, intraocular pressure, and the position of the upper eyelid of the left eye were normal. No signs of conjunctival hyperemia were present. The tumor presented as a yellowish-pink mass with hair and veins on the surface.

**Diagnose:**

The patient was initially diagnosed with a keratoconjunctival tumor of the left eye by a clinical doctor.

**Interventions:**

Corneal tumor resection combined with keratoplasty was performed in the patient. Eye drops with 1% cyclosporine were administered 3 times per day to prevent immune rejection.

**Outcomes:**

Based on postoperative pathological examinations, the final diagnosis was a corneal dermoid. The patient had an uneventful healing process and rapid corneal re-epithelization. The ocular surface was stable during the follow-up visits, and no complications emerged.

**Lessons:**

We report a rare case of congenital corneal dermoid. We learned that close follow-up is needed after surgery in such cases.

## 1 Introduction

Corneal dermoids are congenital benign tumors that usually manifest in the subtemporal quadrant of the eye. They present as pale or yellow semi-circular masses with a keratinized surface and occasionally with hair follicles and cilia. Associated ocular abnormalities include eyelid colobomata, lacrimal anomalies, and scleral and corneal staphylomata.^[1]^ As the tumor grows, patients become impaired by astigmatism, encroachment on the visual axis, and fatty acid infiltration into the cornea. The lesion generally invades the superficial layer of the cornea, though occasionally involves the entire corneal thickness and the anterior chamber. Different surgical techniques for dermoid removal have been described in the past decades, including bare excision, amniotic membrane transplantation, and lamellar or penetrating keratoplasty.^[1,2]^

Keratoplasty is used to improve sight, relieve pain, and treat severe infection or damage. The choice of treatment depends on the tumor grading system based on the regions involved, as described by Mann.^[3]^

The treatment of corneal dermoids is particularly challenging in young children because of several concerns, such as the tendency for low visual acuity and astigmatism-related amblyopia in this population. Recently, a young child with a congenital corneal dermoid was admitted to our department. The manifestation, treatment, and prognosis of this patient are reported below.

## 2 Patient information

The patient was a 1-year-old girl. Her parents had noticed a mass on the surface of the left eyeball that had been present since birth. The patient had no history of other diseases, no relevant family history, and no genetic diseases.

## 3 Clinical findings

**Figure 1:**
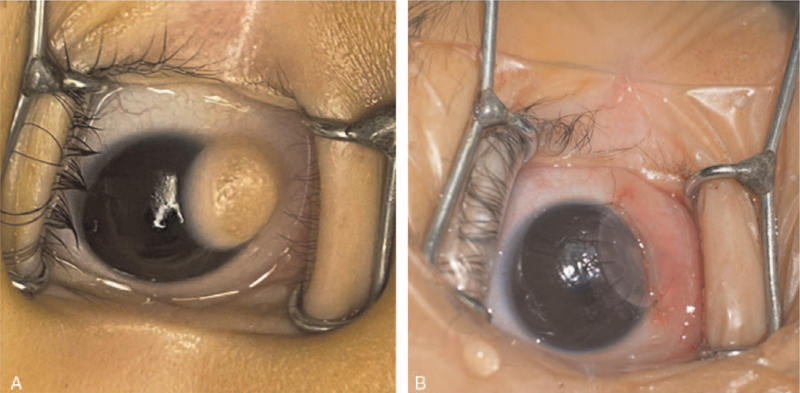
A. Clinical photograph of the cornea before the surgery. B. Clinical photograph of the cornea after corneal transplantation.

Physical and eye examinations were performed when the patient was admitted to the hospital. Eye movement and intraocular pressure in both eyes were normal. The upper left eyelid was in the correct position; mass compression signs were apparent at the eyelid margin, and the mass was slightly exposed when the eyelids were closed. No evident conjunctival hyperemia was noted; however, a small round mass was visible in the 4 to 5 o’clock direction of the temporal cornea. The tumor size was approximately 7 mm × 5 mm × 5 mm, and the appearance was yellowish pink. Hair and veins were also observed on the mass surface. The tumor had clear boundaries, and the conjunctiva was invaded. The remaining cornea was transparent, with no apparent abnormalities in the anterior segment (Fig. 1A). The optic disc boundary was conserved and with an orange hue. Examination of the anterior and posterior chambers of the right eye showed no abnormalities.

Due to the young patient's age, the surgery required general anesthesia, and standard preoperative tests were performed. Orthogonal radiographic chest examination showed that the lungs had a clear texture and no abnormalities. There were also no abnormalities on routine blood examinations, virus screening, or tests for blood clotting disorders.

## 4 Diagnostic assessment

The patient was diagnosed with a keratoconjunctival tumor of the left eye by a clinical doctor based on the appearance and location of the mass.

## 5 Therapeutic intervention

Before the operation, the treatment methods, postoperative precautions, and prognosis were discussed with the patient's parents, and consent was obtained before the operation. Corneal tumor resection combined with keratoplasty was performed under general anesthesia. The superior rectus muscle was fixed using a 5 to 0 nylon wire. The bulbar conjunctiva surrounding the mass was cut to expose the tumoral tissue completely. The tumor and turbid cornea were removed, and a lamellar keratectomy was performed on 1/3 of the thickness of the cornea.

During the operation, a trephine, with a diameter 0.25 mm larger than the implantation bed, was used to drill the corneal graft at the appropriate depth; micro scissors were used to trim the edge and thickness of the graft to fit the implantation bed. The graft was carefully spread onto the implantation bed. The corneal and sclerotic sides were diagonally sutured with 10 to 0 nylon sutures, and the whole graft circumference was intermittently and evenly sutured. At the end of the operation, the corneal graft was smooth and fitted adequately in the implantation bed (Fig. 1B). The sutures did not cause obvious traction on the cornea. The tumor tissue was sent for pathological examination.

## 6 Follow-up and outcomes

**Figure 2:**
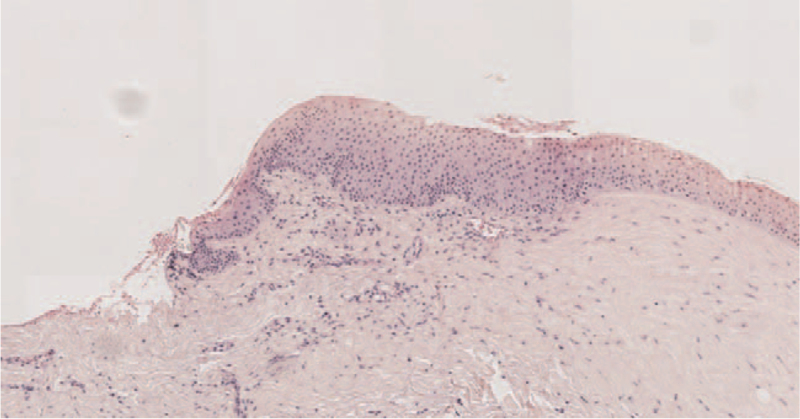
Microphotograph of the excised mass. The surface is composed of stratified squamous epithelium, while the subepithelial fibrous tissue is degenerated hyaline, and localized adipose infiltration is visible (Hematoxylin-eosin, 4 × 10).

In the postoperative pathological examination, the surface of the tumor was found to be composed of stratified squamous epithelium. The subepithelial fibrous tissue was degenerated hyaline, and localized adipose tissue could also be observed (Fig. 2). Based on these findings, the final diagnosis was of a corneal dermoid, classified as grade II based on the grading system created by Mann.^[3]^

One week after the surgery, the corneal epithelium of the graft had grown satisfactorily. The patient was administered 1% cyclosporine eye drops 3 times per day to prevent immune rejection. The parents were urged to prevent the patient from rubbing her eyes, which would cause the surgical suture to dislodge and affect the operation prognosis. During the follow-up, the eye examinations showed no abnormalities. In the third month after surgery, some sutures started to become loose and were removed. All sutures were completely removed 6 months after the surgery. After suture removal, the patient had an excellent recovery.

## 7 Discussion

The patient reported here had an excellent outcome after surgery, mainly because the surgery was performed timely. If the surgery were performed after the tumor became too enlarged and invasive, it would have involved more healthy corneal tissues, thus affecting the recovery, and the outcome would have been worse. We used allograft tissue for the transplantation in this patient; this practice has the advantage of mild postoperative adverse reactions; however, few donors are generally available, and organs are in short supply. With this patient, we could monitor and record the whole case progression, from the disease presentation to surgery and follow-up. This opportunity allowed us a clearer understanding of this disease, with strong clinical implications that may improve treatments for corneal dermoids in children. However, this study has some limitations. Due to insufficient tissue samples, we could not perform further research about this case, such as sequencing the tumoral cells and comparing them with the normal tissues. More studies regarding congenital corneal dermoid are needed.

Corneal dermoids are unusual congenital anomalies formed by residual primitive tissues. These formations are the most common congenital orbital lesions and account for 25% of invasive orbital growths and 75% of cystic lesions.^[1,2]^ Their incidence among newborns is approximately 1 to 3/100,000, and they are equally distributed between sexes.^[1]^ A study by Nevares et al^[4]^ indicated that most ocular dermoids (76%) occur at the inferotemporal bulbar location. However, these masses may occasionally be present entirely within the perilimbal cornea or confined to the conjunctiva and sclera. Limbal dermoids mainly include 4 pathological types: dermoids, lipodermoids, complex choristomas, and epibulbar osseous choristomas.^[5]^ Most of these tumors manifest as dermoids containing pilosebaceous structures, stratified squamous epithelium, and choristomatous tissue, a dense connective tissue similar to the skin corium.

Corneal dermoids are classified into 3 major grades based on their size, anatomical location, and the severity of symptoms they cause.^[3]^ Among grade I tumors, the most frequent are the limbal or epibulbar dermoid types. Grade I tumors are less than 5 mm in size and are usually located in the inferior temporal quadrant of the corneal limbus. They present as a round, flattened, yellowish-pink mass not affecting the entire cornea. Currently, the standard medical approach for grade I disease is observation. Grade II tumors comprise large limbal dermoids that can affect the corneal stroma or Descemet's membrane. Most patients diagnosed with these tumors have difficulties in closing their eyes. Grade III tumors are the rarest and most serious type. They may affect the entire corneal tissue, damage the anterior chamber structure, and occasionally invade the lens.^[3]^

Overall, corneal dermoids may cause visual abnormalities by the gradual development of corneal astigmatism, caused by encroachment in the visual axis, and by corneal infiltration with fatty components. These abnormalities may result in anisometropic amblyopia. The invaded angular axis of the dermoid is the most significant factor associated with postoperative visual acuity, amblyopia development, and postoperative scarring.^[6]^ Furthermore, approximately 30% of patients with corneal dermoids have other congenital abnormalities, the most common being Goldenhar–Gorlin syndrome.^[6]^

Due to their location on the eye surface, corneal dermoids can be easily detected early; thus, most patients can receive timely diagnosis and treatment. Removal of corneal dermoids can lead to persistent epithelial defects, corneal vascularization, and scar formation. All these complications are believed to occur because of focal marginal limbal stem cell deficiency at the site of dermoid excision^[7]^; thus, stem cell transplantation can be performed to minimize the incidence of postoperative defects. In fact, in a recent study, researchers performed amniotic membrane transplantation to remove limbal dermoids and obtained promising postoperative results.^[8]^ Autologous limbal stem cell transplantation has also been successfully implemented in removing corneal dermoids in 2 patients without any of the complications above and no visual loss.^[7]^ Therefore, at present, the primary treatment for this disease is surgical resection combined with corneal or stem cell transplantation. For example, surgical approaches for grade II and III limbal dermoids include excision, lamellar or penetrating keratoplasty, and amniotic membrane transplantation.^[9–11]^ Customized preoperative treatment plans are also available and have been proposed depending on the characteristics of the lesion and the extent of invasion.^[11]^

In conclusion, we reported the rare case of a young patient diagnosed with congenital corneal dermoid. A prompt surgery was performed, resulting in a positive outcome; this case helps us acquire increased knowledge of this disease. Complications such as corneal scarring, neovascularization, pseudopterygium, and eyelid adhesion are common with simple surgical excision. Therefore, we chose to perform tumor resection combined with lamellar keratoplasty for this patient. However, such a procedure is not devoid of postoperative complications, including corneal astigmatism, rejection, and implantation bed penetration. Therefore, we will follow-up with this patient with a complete optical inspection when the child's visual system is fully developed. Refractive correction should be performed to avoid amblyopia if the astigmatism is severe.

## Author contributions

**Data curation:** Yanyan Cui, Shan Yin, Bojun Zhao.

**Formal analysis:** Yanyan Cui, Shan Yin.

**Investigation:** Yanyan Cui.

**Resources:** Xuewei Yin.

**Software:** Yanyan Cui, Shan Yin, Xuewei Yin.

**Supervision:** Xuewei Yin, Yonghui Liu.

**Visualization:** Yonghui Liu, Bojun Zhao.

**Writing – original draft:** Yanyan Cui.

**Writing – review & editing:** Yanyan Cui, Bojun Zhao.
